# Real-world data on uptake and use of digital mental health interventions among waitlisted patients with various mental disorders

**DOI:** 10.1016/j.invent.2026.100915

**Published:** 2026-02-13

**Authors:** Marie Neubert, Esra Sünkel, Jari Planert, Anne Hildebrand, Tim Klucken

**Affiliations:** a Department of Clinical Psychology and Psychotherapy, University of Siegen, Germany

**Keywords:** Digital mental health intervention, Psychological intervention, Outpatient psychotherapy, Real-world data, Naturalistic study, Mental disorders, Psychological well-being

## Abstract

**Background:**

Digital mental health interventions (DMHI) have the potential to provide patients awaiting outpatient psychotherapy with a valuable and immediate treatment option to bridge long waiting periods. However, randomized controlled trials concerning these interventions are marred by high attrition rates and low adherence, and research on the implementation of these interventions in real-world settings is scarce. The present study aims to provide real-world data on the uptake and use of prescribed DMHI, as well as the effect of DMHI use on psychological well-being.

**Method:**

150 patients with various mental disorders awaiting face-to-face psychotherapy were included in this preregistered study. All patients received a prescription for a diagnosis-specific DMHI to bridge the waiting period. Structured telephone interviews were conducted to assess uptake of the prescription, use of the DMHI, and psychological well-being four and 12 weeks after inclusion.

**Results:**

56% of the patients reported an uptake of the DMHI prescription. The percentage of patients who actually used the DMHI was lower than for uptake (29%). Patients who expressed interest in the use of DMHI, higher treatment expectations, and lower psychological well-being were more likely to use the DMHI. A linear mixed model indicated a significant improvement in psychological well-being among DMHI users.

**Conclusion:**

The findings of the present study underscore that the implementation of DMHI in real-world settings is hindered by low uptake rates and even lower utilization. To improve DMHI use, it is essential to incorporate potential mediating factors, such as treatment expectations, into future research.

## Highlights

• Utilization of digital mental health interventions (DMHI) is low in the real world.
• Treatment outcome expectations and utilization of DMHI are linked.
• It is not just access to DMHI that is beneficial for well-being, but its actual use.

## 1 Introduction

Globally, around one in five individuals (18%) experiences a mental disorder within a 12-month period, and the lifetime prevalence for mental disorders is even higher, at approximately 29% (Steel et al., 2014). During the COVID-19 pandemic, the prevalence rates of mental disorders increased substantially, with meta-analytic estimates indicating a prevalence of 34% for depression and 30% for anxiety in the general population (Chekole and Abate, 2021). Consistent with these findings, the 2021 Global Burden of Disease Study 2021 reported a significant increase in depression and anxiety (Murray, 2024). In light of the substantial impact of mental disorders on general human health and economic losses, ensuring adequate treatment for these conditions appears essential (Chekole and Abate, 2021). However, in most countries worldwide, there remains an unmet need and limited access to professional mental healthcare services (Holmes et al., 2018; Walker et al., 2015).

Digital interventions for the treatment of mental disorders represent a promising approach that has the potential to address this gap in mental healthcare (Kazdin, 2017). There is a growing body of research that supports the effectiveness of digital mental health interventions (DMHI) delivered via mobile applications or web-based platforms in addressing common mental disorders. Numerous studies and meta-analyses have indicated the efficacy of various DMHI in reducing symptoms associated with prevalent mental disorders, with a particular focus on digital interventions grounded in cognitive behavioral therapy (e.g., Harrer et al., 2025; Linardon et al., 2019, Linardon et al., 2020; Lindsay et al., 2024; Liu et al., 2024). For example, meta-analytic findings demonstrate that the use of DMHI substantially reduced depressive symptoms and anxiety compared to wait-list controls (e.g., Linardon et al., 2025a; Ma et al., 2021; Pauley et al., 2021). Moreover, the impact on depressive symptoms appears to be consistent, as demonstrated by a reduction in symptoms observed at follow-up assessments up to 12 months (Fuster-Casanovas et al., 2025). Nonetheless, there are several challenges associated with DMHI, including inadequate data management, safety concerns regarding adverse events or harmful content, and insufficient regulatory oversight in public app stores (Huckvale et al., 2020). In order to overcome the aforementioned challenges, there is a call to implement systems for evaluating and regulating DMHI (Fairburn and Patel, 2017).

In 2019, Germany implemented the Digital Healthcare Act (Digitale-Versorgung-Gesetz), becoming one of the first countries to establish a regulatory framework for digital health interventions to treat and manage mental disorders as well as medical issues such as diabetes (Brönneke et al., 2023). The Federal Institute for Drugs and Medical Devices (Bundesinstitut für Arzneimittel und Medizinprodukte; BfArM) is responsible for maintaining a directory in which approved digital health interventions are officially listed. To be included in the directory, and to overcome the aforementioned challenges, the manufacturers of digital health interventions are required to provide high standards of data management and protocols for adverse events. Moreover, the manufacturers are obligated to demonstrate a positive healthcare effect in at least one clinical trial (Mäder et al., 2023). All applications listed in the directory will be reimbursed by health insurance providers and can be prescribed by medical doctors or trained psychotherapists (Goeldner and Gehder, 2024). Approximately 50% of all listed digital interventions in the directory fall within the domain of mental health, with positive treatment effects for depression, anxiety disorders, eating disorders, somatoform disorders, addiction, and borderline personality disorder (Assmann et al., 2025; Hildebrand et al., 2025; Krämer et al., 2022; Planert et al., 2025; Pruessner et al., 2024).

Despite the potential benefits of the regulated implementation of digital health interventions in Germany, clinical trials concerning the efficacy of these interventions are marred by high rates of attrition and the possibility of publication and selection bias (Kolominsky-Rabas et al., 2022; Linardon et al., 2025b). First, in consideration of attrition, previous studies have shown that dropout rates in randomized clinical trials (RCT) on DMHI are generally high (Linardon and Fuller-Tyszkiewicz, 2020). For instance, a meta-analysis of dropout rates in clinical trials of smartphone applications to treat depression revealed a mean dropout rate of 48% after adjusting for publication bias (Torous et al., 2020). An examination of real-world data concerning user engagement with unguided mental health applications downloaded from general app stores, indicated that attrition is even more substantial than in clinical trials, with a median retention rate of 3.9% 15 days following installation (Baumel et al., 2019). Secondly, regarding the possibility of bias, most studies indicating a positive treatment effect for a listing in the official directory of the German BfArM were conducted by the manufacturers or with the participation of manufacturers (Schreiter et al., 2023). Additionally, the possibility of selection bias is especially high in the context of DMHI research, since patients who participate in these clinical trials tend to be those who are already expressing a high level of interest in digital treatment options. Recent findings from a large-scale population-based survey suggest that acceptance rates of DMHI in the general population are relatively low. The survey revealed that 46% of the participants indicated a lack of willingness to use a DMHI for addressing future psychological problems (Steubl et al., 2025). To the best of our knowledge, there has been a scarcity of independent research on the effectiveness of most of DMHI listed in the German directory. Moreover, there is a necessity for research on the implementation of these interventions in real-world settings, since the majority of evidence originates from clinical trials (Brönneke et al., 2023).

In light of the scarcity of independent research on the effectiveness of most DMHI listed in the German directory, coupled with the high selection bias present in clinical trials on DMHI, our study is among the first to examine the uptake and use of a variety of prescribed DMHI in a naturalistic, real-world setting. Furthermore, the present study aims to explore potential factors associated with the uptake and use of a prescribed DMHI, and how uptake and use affect psychological well-being under real-world conditions. To do so, a diagnosis-specific DMHI was prescribed to all patients who were waitlisted for face-to-face psychotherapy in an outpatient clinic. This naturalistic setting was chosen to prevent the aforementioned selection bias. Following a four-week period, and again after 12 weeks, patients were interviewed with questions relating to their psychological well-being, as well as the uptake and use of the prescribed DMHI. The decision to assess the uptake of the prescription by submitting it to the health insurance company, and the use of the DMHI separately, was made to capture various phases in which barriers to the utilization of a DMHI may emerge under real-world conditions. Based on the existing literature, which indicates high attrition rates even in selective samples within clinical trials, we hypothesized that there would be differences between patients in uptake and use of the prescribed DMHI in this real-world setting. Moreover, we hypothesized that the use of the DMHI under real-world conditions would improve the psychological well-being of users compared to non-users.

## 2 Method

### 2.1 Participants

**Fig. 1:**
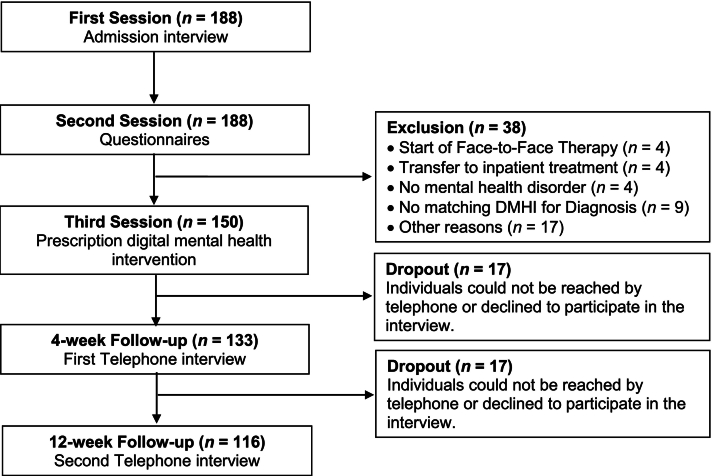
Study procedure and CONSORT flow diagram for participants. This figure illustrates the study procedure and reasons for exclusion and dropout during the procedure. Telephone interviews were conducted approximately 4 and 12 weeks after patients received a prescription for a diagnosis-specific digital mental health intervention (DMHI).

In total, 188 patients were initially screened for study participation. To take part in this study, patients had to be at least 18 years old and had to be seeking face-to-face psychotherapy in an outpatient setting. All eligible 188 patients were invited to the psychotherapeutic outpatient clinic of the University of Siegen. After further screening, a total of 150 patients were included in this study (see Table 1 for demographic information). Data were collected from January 2024 to April 2025. The project has been registered on the Open Science Framework (Neubert and Klucken, 2024). The power analysis was run with G*Power 3.1.9.7. Assuming a medium effect size of ρ = 0.35, we aimed to collect complete data for all study phases from at least 61 participants for a power of 0.85. The study was approved by the institutional ethics committee at the University of Siegen. All participants provided written informed consent. Fig. 1 summarizes the study procedure, reasons for exclusion after the initial screening, and study participation over the course of the study.

**Table 1:** Descriptive statistics of study variables over the course of the study.

Variables	Values
T1: Study entry (N = 150)	
Age in years, *M* (SD)	35.8 (13.9)
Females, *N* (%)	102 (68.0)
Education, *M* (SD)	3.3 (1.5)
Psychotropic medication T_1_, *N* (%)	55 (36.9)
Interest in DMHI use, *N* (%)	85 (57.4)
Positive treatment expectation, *N* (%)	75 (55.6)
Attitudes towards DMHI, *M* (SD)	44.2 (7.8)
Diagnosis-specific DMHI, N (%)	
Unipolar depression	98 (65.3)
Agoraphobia & panic disorder	16 (10.7)
Social anxiety disorder	17 (11.3)
Generalized anxiety disorder	3 (2.0)
Bulimia nervosa	5 (3.3)
Borderline personality disorder	4 (2.7)
Others	7 (4.7)
Psychological well-being T_1_, *M* (SD)	8.2 (4.6)

T2: 4-week follow-up telephone interview (N = 133)	
DMHI uptake, *N* (%)	75 (56.4)
DMHI use, *N* (%)	39 (29.3)
Psychotropic medication T_2_, *N* (%)	52 (39.1)
Psychotherapeutic treatment T_2_, *N* (%)	4 (3.0)
Psychological well-being T_2_, *M* (SD)	9.5 (4.9)

T3: 12-week follow-up telephone interview (N = 116)	
DMHI use after 4-week follow-up, *N* (%)	60 (52.2)
Psychotropic medication T_3_, *N* (%)	49 (42.2)
Psychotherapeutic treatment T_3,_*N* (%)	21 (18.1)
Psychological well-being T_3_, *M* (SD)	10.3 (5.4)

*Note.* The table represents all study variables and outcomes measured at three different times over the study course. Sociodemographic variables were measured at study entry (T_1_). Uptake and use of digital mental health interventions (DMHI) were measured at a 4-week follow-up (T_2_) as well as a 12-week follow-up (T_3_).

### 2.2 Study procedure

All eligible patients, who were invited to the psychotherapeutic outpatient clinic, attended three sessions with a trained psychotherapist. In the first session, the psychotherapist conducted an admission interview, during which they conducted a comprehensive evaluation of the patients' symptoms and addressed the patients' symptom history as well as previous treatments. In the second session, patients were asked to provide sociodemographic data and complete several questionnaires. These included both outcome- (e.g., psychological well-being) and process-related (e.g., attitudes towards psychological online interventions) instruments. Following this, the trained psychotherapists diagnosed patients' mental disorders on the basis of structured clinical judgment. The third session comprised an explanation of the patient's mental disorder and the available treatment possibilities. In cases where patients were placed on a waiting list for face-to-face psychotherapy and a diagnosis-specific DMHI that was suitable for their particular mental disorder was available, psychotherapists recommended and prescribed this DMHI. All psychotherapists were instructed to deliver a standardized recommendation to use the DMHI (i.e., “*DMHI are digital interventions to overcome waiting periods for face-to-face psychotherapeutic sessions. Medical doctors and psychotherapists are allowed to prescribe DMHI. I recommend that you use the prescribed DMHI for [diagnosis], so that you receive treatment as soon as possible.”*). Furthermore, psychotherapists handed out an information sheet detailing the specifics of DMHI, the process of securing access to the prescribed digital treatment, and the regulations surrounding data protection. Additionally, the patients were informed that the prescription would only be valid for a period of four weeks, and that they were advised to submit it at the earliest opportunity. At the close of the third session, patients were notified that they would receive a telephone call from a study assistant who would ask about their well-being and their extended search for face-to-face therapy a few weeks from the present date onwards. In order to avoid any influence of the announced interview on uptake and use of DMHI, patients were not informed that the study assistant would ask about the DMHI. The follow-up telephone interviews took place approximately 4 and 12 weeks after the psychotherapist handed out the prescription for the diagnosis-specific DMHI in the third session. The telephone interviews were conducted by trained study assistants using a semi-structured interview. The semi-structured interview, which lasted approximately 20 min, entailed questions regarding uptake and use of the DMHI, satisfaction and obstacles with the digital treatment, as well as patients' psychological well-being.

### 2.3 Digital mental health interventions (DMHI)

In this study, psychotherapists could prescribe one out of 11 pre-selected DMHI for the following primary diagnoses: unipolar depression, agoraphobia and panic disorder, social anxiety disorder, generalized anxiety disorder, chronic pain disorder, bulimia nervosa, binge-eating disorder, borderline personality disorder, insomnia, harmful use and addiction of alcohol, and burnout. In the course of the study's planning phase, 11 DMHI were selected in advance from a total of 25 DMHI for mental disorders, as listed in the directory for DMHI of the BfArM. For a number of diagnoses, a multitude of DMHI were listed by various providers (e.g., six DMHI for unipolar depression). In order to minimize variations in the prescriptions made by the psychotherapists in this study, 11 DMHI, one for each mental disorder, were pre-selected. In the selection process, DMHI that had been accepted on a permanent basis were given priority over those DMHI that had been provisionally accepted. The acceptance of a DMHI on a permanent basis is dependent on the demonstration of a positive treatment effect in at least one RCT. Secondly, app-based applications were preferred over web-based applications. Previous research suggests that app-based applications are more intuitive and user-friendly, and may therefore improve user engagement (Purkayastha et al., 2020). Of the 11 selected DMHI, four only provided a web-based user environment, while nine provided an app-based application or both. In instances where multiple DMHI were identified for a particular disorder following the initial selection process, the DMHI that had been most frequently prescribed by psychotherapists and general practitioners in the past was selected.

### 2.4 Assessments

#### 2.4.1 Sociodemographic and patient-specific characteristics

Patients provided information on their sociodemographic characteristics (i.e., age, sex, and education) and patient-specific characteristics (i.e., psychotropic medication, interest in DMHI use, treatment expectation, and attitudes towards DMHI) during the first two sessions at the outpatient clinic. Sociodemographic characteristics were assessed during the admission interview. The patient's highest level of general education was determined using a 7-point scale, where higher scores indicate a higher level of education. Level one includes individuals with secondary school diplomas after nine years of schooling, while level two includes individuals with secondary school diplomas after ten years of schooling. Level three comprises individuals with a technical college entrance qualification, while level four includes individuals with a general university entrance qualification. Level five comprises individuals with a bachelor's degree, while level six encompasses those with a master's degree. The highest level comprises individuals with a doctoral degree.

The current use of psychotropic medication was also evaluated by a trained psychotherapist during the admission interview in the first session. Patients were asked to provide the name of the medication and the current prescribed dosage. For data analysis, a dichotomous variable was generated to reduce complexity. This variable had “0” representing no current use of psychotropic medication and “1” for current use of medication.

Interest in DMHI use, treatment expectation, and attitudes towards DMHI were assessed using a paper-and-pencil questionnaire before the admission interview. Interest in DMHI use was measured by a single dichotomous item (i.e., “*Would you be interested in using psychological online interventions?*”) with “0” representing no interest and “1” representing interest in DMHI use. Furthermore, treatment expectancy was measured by a single dichotomous item (i.e., “*Do you think that psychological online interventions can help you with your mental health problems?*”) with the response format yes = 1 and no = 0.

Attitudes towards DMHI were assessed using the Attitudes towards Psychological Online Interventions (APOI) questionnaire for patients (Schröder et al., 2015). The APOI consists of 16 statements about psychological online interventions, and respondents rate their agreement with each statement on a 5-point scale ranging from 1 (totally disagree) to 5 (totally agree). Four subscales (i.e., skepticism and perception of risks, confidence in effectiveness, technologization threat, anonymity benefits). A total score can be calculated, with higher scores reflecting more positive attitudes towards psychological online interventions. According to the authors, the internal consistency and validity of the questionnaire were good (Schröder et al., 2015). We also found a good internal consistency for the total score in the current sample (Cronbach's α = 0.81).

#### 2.4.2 Psychological well-being

Psychological well-being was assessed using the World Health Organization Well-Being Index (WHO-5). The WHO-5 is a short questionnaire comprising five items, which can be used for self-assessment or administered orally. Respondents rate their psychological well-being over the previous two weeks on a scale ranging from 5 (all of the time) to 0 (at no time). Higher scores indicate higher psychological well-being. In this study, the WHO-5 was self-administered during the third session at the outpatient clinic and during follow-up telephone interviews at four and 12 weeks after the third session using oral administration. The WHO-5 is a widely used and comprehensive outcome measure in clinical trials as well as a screening tool for depression with adequate validity (Topp et al., 2015). Internal consistency for the WHO-5 in the current sample was good (Cronbach's α = 0.84).

#### 2.4.3 Structured telephone interviews

Semi-structured interviews were conducted by trained study assistants approximately four and 12 weeks after the third session at the outpatient clinic. The interviews were conducted via the telephone and lasted approximately 20 min. The interview addressed aspects of uptake, use, and user satisfaction regarding the prescribed DMHI. The assessment of uptake was conducted solely at the four-week follow-up, as the prescription for a DMHI is valid for a period of only four weeks. Patients were also asked if they had received other treatment since the third session (i.e., psychotropic medication, outpatient clinic visits, inpatient clinic stays). Furthermore, respondents were asked to provide information about their psychological well-being over the previous two weeks using the WHO-5 scale. In order to assess the uptake of the DMHI, patients were asked to indicate whether and when they submitted the prescription to their healthcare provider, as well as the number of days taken to receive an access code to activate the DMHI. In addition, patients were asked whether they had activated the DMHI and if they had used it after activation. In the event that patients did not use the DMHI, study assistants explored the reasons for this non-adoption. Patients who used the DMHI also responded to questions regarding their satisfaction.

User satisfaction was assessed orally using the client satisfaction questionnaire (CSQ—I), a tool specifically adapted for the context of internet-based health interventions (Boß et al., 2016). The CSQ-I comprises eight items, and patients were asked to rate each item on a 4-point scale ranging from 1 (does not apply to me) to 4 (totally applies to me). The total score ranges from eight to 32, with higher scores indicating higher satisfaction. The questionnaire has been demonstrated to be a valid and reliable measure to capture user satisfaction with internet-based health interventions (Boß et al., 2016). The current sample displayed good internal consistency for the CSQ-I (Cronbach's α = 0.83).

### 2.5 Statistical analyses

Statistical analyses were carried out with IBM SPSS version 31.0 for Windows (Chicago, SPSS, Inc.). All study variables were screened for extreme univariate outliers using boxplots. Occurring extreme outliers were checked for plausibility. As a consequence of dropout, some missing data occurred. Statistical significance was set at a *p-*value ≤0.05 for all analyses*.*

To analyze associations of sociodemographic (e.g., age, sex, education), patient-specific (e.g., psychotropic medication), and psychological (e.g., well-being, treatment expectation) variables with uptake and use of the prescribed DMHI, bivariate correlation analyses were used.

The effects of uptake and use of the prescribed DMHI on psychological well-being at 4- and 12-week follow-up were examined using linear mixed models with restricted maximum likelihood estimation. Linear mixed models are recommended when dealing with missing data in the analysis of repeated measures (Detry and Ma, 2016; Magezi, 2015). Initially, the effects of uptake (group variable: yes/no) and use (group variable: yes/no) of the DMHI on well-being were analyzed using separate models. Subsequently, psychotropic medication and current psychotherapeutic face-to-face treatment were entered into the analyses as covariates. To select the best-fitting model for each analysis, different covariance structures were tested. The lowest Bayesian information criteria (BIC) was chosen to indicate the best model fit. All models included group as a factorial predictor, time as a continuous predictor (study entry = T1, 4-week follow-up = T2, 12-week follow-up = T3), a group x time interaction, a random intercept, and a random slope.

## 3 Results

A total of 150 patients were included in the present study and received a prescription for a diagnosis-specific DMHI. Missing values occurred during assessments as follows: Education (1.3%), Psychotropic medication (0.9%), Interest in DMHI use (1.3%), positive treatment expectation (10.0%), APOI (8.7%), WHO-5 T_1_ (6.7%), DMHI use after 4-week follow-up (0.9%), and WHO-5 T_3_ (0.9%). Table 1 shows descriptive statistics for all study variables at study entry, 4-week follow-up, and 12-week follow-up.

### 3.1 Uptake and use of the prescribed digital mental health intervention (DMHI)

The uptake and use of the prescribed DMHI were assessed four and 12 weeks after the patients received their prescription. At the 4-week follow-up, 75 patients of the 133 patients available (56.4%) submitted the prescription for the DMHI to their health insurance company for an activation code. 48 patients of the 133 (36.1%) used the activation code to install and activate the DMHI. However, only 39 of the 133 patients (29.3%) reported using the DMHI. Those who used the DMHI were asked about their user experiences. The majority of patients, 26 out of 39 patients (66.7%), reported that the use of the DMHI was beneficial. Patient satisfaction measured using the CSQ-I (score range: 8–32), was found to be generally high, with a mean (*SD)* satisfaction score of 24.2 (4.8). Moreover, 37 patients (94.9%) wished to continue the use of the DMHI. At the 12-week follow-up, 116 of the 150 patients initially included in this study were reached for the second telephone interview. When asked about their use of the DMHI since the first follow-up telephone interview, at least 60 of the 116 patients (52.2%) reported having used the DMHI. This time, 47 patients out of 60 (78.3%) expressed that the use of the DMHI was beneficial. Patient satisfaction scores were found to be consistent with those observed at the 4-week follow-up, with a mean (*SD)* satisfaction score of 24.3 (6.3).

Associations of sociodemographic, patient-specific, and psychological variables with uptake and use of the prescribed DMHI are presented in Table 2. Bivariate correlation analyses revealed that DMHI uptake at the 4-week follow-up telephone interview was significantly associated with the use of prescribed psychotropic medication at study entry (*r*(132) = 0.18, *p* = 0.04). Furthermore, patients expressing interest in the use of a DMHI (*r*(132) = 0.20, *p* = 0.02) as well as those patients who expected a positive treatment outcome from the use of a DMHI (*r*(121) = 0.21, *p* = 0.02) were more likely to submit the prescription to their health insurance company to receive access to the DMHI. Uptake of DMHI was also found to be significantly associated with lower psychological well-being at study entry (*r*(124) = −0.23, *p* = 0.01) and reduced psychological well-being at the 4-week follow-up assessment (*r*(133) = −0.30, *p* < 0.001).

**Table 2:** Bivariate associations (r) with the uptake and use of a digital mental health intervention.

Variable	1.	2.	3.	4.	5.	6.	7.	8.	9.	10.	11.	12.
1. Age	**–**											
2. Sex	0.09	**–**										
3. Education	−0.30^⁎⁎^	−0.10	**–**									
4. Psychotropic medication, T_1_	0.16^+^	0.05	−0.08	**–**								
5. Interest in DMHI use	0.08	0.06	0.09	0.00	**–**							
6. Positive treatment expectation	0.08	0.10	0.13	−0.00	0.73^⁎⁎^	**–**						
7. Attitudes towards DMHI	0.04	0.07	0.21^⁎^	−0.04	0.49^⁎⁎^	0.64^⁎⁎^	**–**					
8. Psychological well-being, T_1_	−0.18^⁎^	−0.00	0.19^⁎^	−0.13	0.04	0.11	0.21^⁎^	**–**				
9. DMHI uptake, T_2_	0.13	−0.12	−0.07	0.18^⁎^	0.20^⁎^	0.21^⁎^	0.07	−0.23^⁎^	**–**			
10. DMHI use, T_2_	0.04	−0.01	0.15^+^	0.04	0.21^⁎^	0.24^⁎⁎^	0.12	−0.10	0.57^⁎⁎^	**–**		
11. Psychological well-being, T_2_	−0.30^⁎⁎^	−0.11	0.19^⁎^	−0.13	−0.04	−0.02	0.09	0.64^⁎⁎^	−0.30^⁎⁎^	−0.20^⁎^	**–**	
12. DMHI use, T_3_	0.22^⁎^	−0.11	0.01	0.11	0.21^⁎^	0.24^⁎^	0.07	−0.19^⁎^	0.57^⁎⁎^	0.36^⁎⁎^	−0.28^⁎⁎^	**–**
13. Psychological well-being, T_3_	−0.23^⁎^	−0.03	0.28^⁎⁎^	−0.03	0.12	0.15	0.17^+^	0.45^⁎⁎^	−0.13	0.05	0.62^⁎⁎^	−0.20^⁎^

*Note.* Digital mental health intervention = DMHI; Sex: 1 = female, 2 = male, Psychotropic medication: 0 = no, 1 = yes, Interest in DMHI use: 0 = no, 1 = yes, Positive treatment expectation: 0 = no, 1 = yes, DMHI uptake & use: 0 = no, 1 = yes. T_1_: Study entry, T_2_: 4-week follow-up telephone interview, T_3_: 12-week follow-up telephone interview, ^+^ = *p* < .1; * = *p* < .05; ** = *p* < .01.

Similar relationships as for DMHI uptake were identified for the use of the prescribed DMHI at the 4-week follow-up telephone interview. Use of the DMHI was significantly associated with interest in the use of a DMHI (*r*(132) = 0.21, *p* = 0.02) and positive treatment expectation (*r*(121) = 0.24, *p* < 0.01). Use of the DMHI was also related to reduced psychological well-being at the 4-week follow-up assessment (*r*(133) = −0.20, *p* = 0.02) but not with psychological well-being at study entry (*r*(124) = −0.10, *p* = 0.29). In addition, the bivariate correlation analyses indicated that higher education tended to be significantly associated with DMHI use four weeks after study entry (*r*(131) = 0.19, *p* = 0.09). No other sociodemographic or patient-specific characteristics were found to be significantly related to the uptake and use of the DMHI four weeks after prescription (all *p*-values >0.1).

Concerning DMHI use after the 4-week follow-up assessment, we also found that patients who were interested in DMHI (*r*(114) = 0.21, *p* = 0.02) and those who expected a positive treatment outcome at the time of study entry (*r*(103) = 0.24, *p* = 0.01) were more likely to report the use of DMHI at the 12-week follow-up assessment. Moreover, DMHI use following the 4-week follow-up was associated with diminished psychological well-being at study entry (*r*(111) = −0.19, *p* < 0.05), at the 4-week follow-up (*r*(115) = −0.28, *p* < 0.01), and at the 12-week follow-up (*r*(114) = −0.20, *p* = 0.03). A notable correlation with age was observed, with older patients tending to report use of the DMHI after the 4-week follow-up (*r*(115) = 0.22, *p* = 0.02). No further significant associations were observed with sociodemographic and patient-specific characteristics (all *p*-values >0.1).

### 3.2 Effect of uptake and use on psychological well-being

The linear mixed model to test the effect of the initial uptake of the DMHI on psychological well-being revealed significant main effects for group (*F*(1, 131.01) = 7.81; *p* < 0.01) and time (*F*(2, 167.98) = 12.57; *p* < 0.01)*,* but no significant group x time interaction (*F*(2, 167.98) = 2.75; *p* = 0.07). Similar results were observed analyzing the linear mixed model controlling for psychotropic medication and face-to-face psychotherapy at the follow-up assessments. Significant main effects were observed for group (*F*(1, 129.64) = 7.70; *p* < 0.01) and time (*F*(2, 179.95) = 5.65; *p* < 0.01)*,* but not for psychotropic medication (*F*(1, 303.53) = 0.28; *p* = 0.60) or for face-to-face psychotherapy (*F*(1, 226.35) = 0.01; *p* = 0.92)*.* As in the original model, no interaction for group x time was found (*F*(2, 164.05) = 2.51; *p* = 0.09). The model estimated means (*SD*) for psychological well-being in patients who did not submit the prescription for the diagnosis-specific DMHI were 9.14 (4.99) at the time of study entry, 11.10 (4.84) at the 4-week follow-up, and 10.98 (5.18) at the 12-week follow-up assessment. Patients who submitted the prescription demonstrated lower psychological well-being at the time of study entry (*M:* 7.09, *SD:* 4.93), at the 4-week follow-up (*M:* 8.21, *SD:* 4.85), and at the 12-week follow-up interview (*M:* 9.88, *SD:* 5.00).

**Fig. 2:**
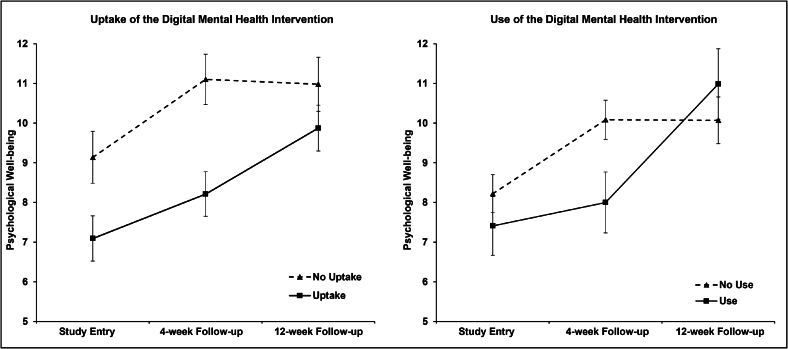
Psychological well-being affected by uptake and use of the digital mental health intervention. Uptake and use of the digital mental health intervention was assessed 4 weeks after patients were handed the prescription for the intervention (4-week follow-up). Values are estimated marginal means (with SEM) from multilevel analysis.

In order to evaluate the impact of the use of the DMHI, which was assessed four weeks after patients received a prescription, on psychological well-being, a linear mixed model was employed. A significant main effect for time (*F*(2, 120.67) = 13.50; *p* < 0.01)*,* but not for group (*F*(1, 128.82) = 0.68; *p* = 0.41) could be identified. Furthermore, a significant interaction for group x time (*F*(2, 120.67) = 6.56; *p* < 0.01) was observed. The model estimated means (*SD*) for psychological well-being in patients who did not use the DMHI were 8.22 (4.63) at the time of study entry, 10.09 (4.79) at the 4-week follow-up, and 10.07 (5.71) at the 12-week follow-up assessment. Patients who used the DMHI reported similar psychological well-being at the time of study entry (*M:* 7.41, *SD:* 4.60) and at the 12-week follow-up interview (*M:* 10.99, *SD:* 4.79), but lower psychological well-being at the 4-week follow-up assessment (*M:* 8.00, *SD:* 5.55). Post-hoc tests for between-group comparisons revealed that patients who used the DMHI, compared to patients who did not, reported significantly lower psychological well-being at the 4-week follow-up assessment, with a mean difference of −2.09 (95% CI: −3.89 to − 0.28, p = 0.02). There was no significant group difference at the time of study entry *(M*_*D*_*:* -0.81; 95% CI: −2.55 to 0.93, p = 0.36)*,* nor at the 12-week follow-up assessment *(M*_*D*_*:* 0.92; 95% CI: −1.20 to 3.03, p = 0.39). Controlling for psychotropic medication and current face-to-face psychotherapy, the second linear mixed model revealed similar results to the first model without covariates. A significant main effect was observed for time (*F*(2, 122.50) = 5.69; *p* < 0.01)*,* but not for group (*F*(1, 126.38) = 0.74; *p* = 0.39)*,* psychotropic medication (*F*(1, 302.59) = 1.04; *p* = 0.31) or for face-to-face psychotherapy (*F*(1, 195.02) = 0.00; *p* = 1.00)*.* As in the first model, a significant interaction for group x time was found (*F*(2, 119.23) = 6.50; *p* < 0.01). Fig. 2 presents the estimated marginal means for psychological well-being at each assessment point for uptake and use of the DMHI assessed at the 4-week follow-up telephone interview.

## 4 Discussion

This study aimed to examine a differentiated perspective on uptake and use of prescribed DMHI, and the effect of DMHI use on psychological well-being during waiting periods for psychotherapeutic outpatient treatment in a real-world setting. As hypothesized, patients differed in their uptake and use of the diagnosis-specific DMHI. Regarding uptake, 56% of the patients submitted the prescription to gain access to the DMHI. The percentage of patients who reported using the DMHI was lower, with only 29% reporting its utilization. Following a 12-week period, 52% of all patients who were reached at the second follow-up reported that they had used the DMHI since the first interview. Patients who expressed an interest in the use of psychological online interventions, higher treatment expectations, and lower psychological well-being were more likely to use the DMHI. Furthermore, the results indicate that the use of the prescribed DMHI during the waiting period for face-to-face psychotherapy enhanced psychological well-being.

The magnitude of patients who did not use the prescribed DMHI seems relatively high, at 48%, but is in line with attrition rates reported in multiple RCTs of psychological online interventions (Torous et al., 2020). However, several studies conducted in collaboration with the manufacturers of DMHI in Germany have reported dropout rates of approximately 25% in the intervention groups, which is considerably lower than the proportion of non-users in our study (Krämer et al., 2022; Mäder et al., 2023). Looking at non-adherence to psychotropic treatment for mental disorders, a recent meta-analysis yielded results consistent with those of the present study, with a prevalence of non-adherence of 46% (Zewdu et al., 2025). The concept of non-adherence encompasses both patients who refuse to take their prescribed medication and those who do not adhere to their treatment plan. Looking only at treatment refusal in RCTs involving psychotropic medication or psychotherapeutic treatment, findings indicate a substantially lower refusal rate of 8% (Swift et al., 2017). This difference may be partially attributed to the fact that RCT participants are, by design, initially willing to engage in treatment. When looking at real-world data for traditional face-to-face psychotherapy, the aggregated attrition rate is 26%, which is significantly lower than the rate for non-use of DMHI in the present study (Swift and Greenberg, 2012). One potential explanation could be the association of treatment preference with dropout and treatment outcomes. Patients who receive a treatment that aligns with their preferences appear to be more likely to complete the treatment and may exhibit better treatment outcomes (Swift et al., 2013). In the present study, patients were seeking face-to-face psychotherapy in an outpatient setting, and only 57% of patients reported interest in DMHI use. Therefore, it is likely that DMHI did not match every patient's treatment preference. In contrast, participants in RCTs conducted in collaboration with the manufacturers of DMHI were predominantly interested in this type of treatment. This phenomenon may provide a rationale for the observation that the dropout rates in these RCTs were considerably lower than the rate of non-users in this study.

The aforementioned explanation of treatment preference is in line with the observation that interest in DMHI use and positive treatment expectations were significantly associated with uptake and use of the DMHI in the present study. As demonstrated by previous research on treatment outcome expectations, positive outcome expectations are associated with better post-treatment outcomes for psychotherapy (Constantino et al., 2018) as well as medical procedures (Rief et al., 2017). Recent studies follow the approach to optimize patients' treatment outcome expectations in order to improve treatment outcomes. The expectation violation model (ViolEx) postulates that the violation of expectations may lead to expectation change, thereby providing a framework for techniques to improve treatment outcome expectations (Rief et al., 2022). The findings from two experimental studies concerning the modification of outcome expectations in psychotherapy indicate that the warmth and competence of therapists are key factors optimizing outcome expectations through expectation violation (Seewald and Rief, 2023, Seewald and Rief, 2024). Future studies should incorporate the assumptions of the ViolEx model to change treatment outcome expectations in DMHI to improve adherence and treatment outcomes.

Improving adherence seems particularly important in light of the present finding, that DMHI use, but not the mere DMHI uptake, seems to enhance psychological well-being at follow-up. This effect still occurred after controlling for psychotropic medication as well as current face-to-face therapy at follow-up assessments. The real-world data presented in this study align with previous findings from RCTs documenting a significant reduction in diagnosis-specific symptoms and elevation of well-being (Assmann et al., 2025; Krämer et al., 2022; Pruessner et al., 2024). In Germany, patients commonly have to wait a minimum of four months or more for face-to-face psychotherapy, and numerous patients experience refusal when seeking outpatient treatment (Zepf et al., 2003). This is particularly relevant when considering evidence that indicates that non-treatment intervals bear the risk of worse outcomes and chronification of pathology (Ghio et al., 2014). It therefore seems relevant to provide patients with effective treatment options like DMHI during waiting periods. However, the findings of this study demonstrate that it is not only important to have access to a DMHI, it is also relevant to use it. It is therefore important for practitioners not only to prescribe a DMHI, but also to explain its benefits and elevate treatment outcome expectations.

Notwithstanding the strengths of this study, such as providing real-world data from a naturalistic outpatient setting and high participation rates in follow-up telephone interviews, the following limitations need to be considered. First, given the naturalistic setting, the generalizability of the results regarding the uptake and use of DMHI for various mental disorders is reduced, since 65% of the included patients received a prescription of a DMHI to treat depressive symptoms, while DMHI to treat other disorders, such as generalized anxiety disorder, were only prescribed for 2% of the patients. A more diverse sample with a more balanced distribution of various mental disorders would allow for the exploration of diagnosis-specific differences in DMHI use. Secondly, the analysis regarding the effect of DMHI use on psychological well-being was only controlled for psychotropic medication and psychotherapy. Future research should focus on moderating and mediating factors when analyzing the impact of DMHI use on psychological outcomes, given the growing body of research investigating predictors of DMHI use. A better understanding of these factors could help to improve treatment outcomes. Third, the use of DMHI was assessed dichotomously. A more differentiated assessment of DMHI use and adherence would allow for a more nuanced analysis of the effectiveness of DMHI use. However, a unified conceptualization to measure adherence in relation to DMHI is currently lacking due to the absence of a concrete definition of intended use, as exists for psychotropic medication (Sieverink et al., 2017). In a recent study, the authors examined the impact of different adherence measures on clinical outcomes in an RCT and found that the predictive value of adherence varied depending on the definition used (Hanano et al., 2022). Fourth, the assessment of uptake and use was solely based on self-report, which may be impacted by socially desirable responses. A further potential limitation of this study is the study design, by including three initial psychotherapy sessions and structured telephone interviews. These study elements may not fully reflect a typical real-world scenario, in which therapeutic contact could be less frequent. Consequently, the data presented in this study may still overestimate uptake and use of DMHI compared to routine care settings.

## 5 Conclusion

This study is among the first to provide real-world data on the multi-step process from prescription to DMHI use in the German health care system, identifying barriers that contribute to its complexity. In conclusion, the results of the present study indicate that the uptake and use of DMHI in a real-world setting is relatively low, with approximately 50% of the patients utilizing this treatment option while waiting for a psychotherapeutic outpatient treatment. Concurrently, our findings indicate that patients who utilized the DMHI during the waiting period reported high levels of satisfaction and showed an enhancement in psychological well-being. It appears that the decisive factor is not the mere access to a DMHI, but rather its actual use. Therefore, future studies should focus on strategies to promote DMHI use and improve clinical outcomes. The findings of this study indicate that treatment outcome expectation might be one key factor in enhancing the use and effectiveness of DMHI.

## Funding

This research did not receive any specific grant from funding agencies in the public, commercial, or not-for-profit sectors.

## Declaration of competing interest

All authors declare that they have no known competing financial interests or personal relationships that could have appeared to influence the work in this paper.
